# Data on proteomic analysis of milk extracellular vesicles from bovine leukemia virus-infected cattle

**DOI:** 10.1016/j.dib.2020.106510

**Published:** 2020-11-10

**Authors:** Md. Matiur Rahman, Yassien Badr, Yuji O. Kamatari, Yuko Kitamura, Kaori Shimizu, Ayaka Okada, Yasuo Inoshima

**Affiliations:** a The United Graduate School of Veterinary Sciences, Gifu University, Japan; b Laboratory of Food and Environmental Hygiene, Cooperative Department of Veterinary Medicine, Gifu University, Japan; c Department of Medicine, Faculty of Veterinary, Animal, and Biomedical Sciences, Sylhet Agricultural University, Sylhet, Bangladesh; d Department of Animal Medicine, Faculty of Veterinary Medicine, Damanhour University, El-Beheira, Egypt; e Division of Instrumental Analysis, Life Science Research Center, Gifu University, Japan; f Gifu Prefectural Chuo Livestock Hygiene Service Center, Japan; g Education and Research Center for Food Animal Health, Gifu University (GeFAH), Japan; h Joint Graduate School of Veterinary Sciences, Gifu University, Japan

**Keywords:** Bovine leukemia virus, Milk extracellular vesicles, NanoLC-MS/MS, Proteomic analysis, Scaffold-DIA

## Abstract

Milk extracellular vesicles (EVs) are nanoparticles that contain proteins, mRNAs, microRNAs, DNAs, and lipids that involved in several biological functions. Milk EVs provide proteins that could represent relevant novel biomarkers for monitoring of different diseases such as breast cancer and mastitis in humans and animals, respectively. Bovine leukemia virus (BLV) is an oncogenic virus that causes progressive B-cell lymphosarcoma in cattle. Here, we aimed to identify proteins in milk EVs from BLV-infected cattle compared with those from uninfcetd cattle. Proteomic analysis was performed by using a comprehensive nano liquid chromatography-tandem mass spectrometry (nanoLC-MS/MS) approach. Identified proteins were analyzed by using a proteomic software, Scaffold-Data Independent Acquisition (Scaffold-DIA).

## Specifications Table

**Table:** 

Subject	Veterinary Medicine
Specific subject area	Proteomic of milk EVs
Type of data	Table and dataset
How data were acquired	NanoLC-MS/MS analysis was used with an UltiMate 3000 RSLC nano HPLC System (Thermo Fisher Scientific, Waltham, MA, USA) coupled with Q Exactive mass spectrometer (Thermo Fisher Scientific).
Data format	Raw and analyzed data
Parameters for data collection	Milk EV proteins from BLV-infected cattle with HPL+HLDH and uninfected cattle
Description of data collection	Milk was collected from three BLV-infected and uninfected cattle followed by isolation of milk EVs. The morphology of milk EVs were observed by TEM analysis. A comprehensive proteomic analysis was performed by using nanoLC-MS/MS method. The data were further analyzed by Scaffold-DIA software (Proteome software, Portland, OR, USA).
Data source location	Institution: Laboratory of Food and Environmental Hygiene, Gifu University. City/Region: Gifu/Gifu Prefecture Country: Japan.
Data accessibility	Repository name: Md. Matiur Rahman Data identification number (BLV-infected cattle): Mendeley Data, v1, DOI: 10.17632/zxb5vhjrf5.1 Direct URL to data: http://dx.doi.org/10.17632/zxb5vhjrf5.1 Data identification number (Uninfected cattle): Mendeley Data, V1, DOI: 10.17632/7c2ddgwcgt.1 Direct URL to data: http://dx.doi.org/10.17632/7c2ddgwcgt.1

EVs, extracellular vesicles; nano-LC-MS/MS, nano liquid chromatography-tandem mass spectrometry; BLV, bovine leukemia virus; HPL, high proviral load; HLDH, high lactate dehydrogenase; TEM, transmission electron microscopy; Scaffold-DIA, Scaffold-Data Independent Acquisition.

## Value of the Data

• The proteomic dataset of bovine milk-derived extracellular vesicles (EVs) will expand the knowledge and create a solid ground for future investigations of bovine leukemia viral (BLV) infection in cattle.
• Identified proteins will give researchers an initial reference database for the understanding of pathogenesis of BLV infection in cattle.
• The bovine milk-derived EV proteomic dataset can be used to classify the possible biological properties of proteins/peptides in bovine milk, or to grant access to useful information for the isolation of desired proteins.

## 1 Data Description

Milk extracellular vesicles (EVs) were isolated from three bovine leukemia virus (BLV)-infected cattle with high proviral load (HPL) along with high percentage of lactate dehydrogenase isozymes (HLDH) 2 + 3 and uninfected cattle (Table 1). The morphology of milk EVs were observed by transmission electron microscopy (TEM) analysis (Data not shown). The proteomic landscape of milk EVs from BLV-infected and uninfected cattle was analyzed by nano liquid chromatography-tandem mass spectrometry (nanoLC-MS/MS) with an Ultimate 3000 RSLC nano HPLC system connected to a Q Exactive mass spectrometry (Thermo Fisher Scientific, Waltham, MA, USA). Identification and quantification of proteins were performed by using Scaffold-Data Independent Acquisition (Scaffold-DIA) software.

**Table 1:** Clinical status of the BLV-infected and uninfected cattle used in this study.

Cattle no.^a^	Age (months)^b^	ELISA^c^	Nested PCR^d^	Proviral load^e^	LDH isozyme^f^					
					1	2	3	2 + 3	4	5
BLV-infected cattle										
1	84	+	+	32,023	47.7	25.1	16.5	41.6	6.7	4.0
2	72	+	+	33,480	54.5	15.6	15.8	31.4	7.7	6.4
3	96	+	+	36,859	58.4	18.8	13.5	32.3	6.3	3.2
Uninfected cattle										
1	108	–	–	NT	63.9	16.6	13.0	29.6	4.3	2.2
2	48	–	–	NT	NT	NT	NT	NT	NT	NT
3	84	–	–	NT	54.2	26.1	13.5	39.6	4.4	1.8

+, positive; -, negative; NT, not tested;.

a no., number;.

b Age at blood sampling;.

c ELISA, enzyme-linked immunosorbent assay;.

d PCR, polymerases chain reaction;.

e copies/10^5^ white blood cells DNA;.

f LDH, lactate dehydrogenase.

## 2 Experimental Design, Materials and Methods

### 2.1 Hematology

#### 2.1.1 Sample collection

Blood was collected from Holstein dairy cattle in vacuum blood collection tubes with or without an anti-coagulant (VP-AS076 K, VP-NA050 K, and VP-H070 K, Terumo, Tokyo, Japan). Plasma was separated by centrifugation with MX-307 (Tomy Seiko, Tokyo, Japan) at 2500 × *g* for 15 min at 25 °C followed by serum separation from clotted blood by centrifugation at 3000 × *g* for 15 min at 25 °C.

#### 2.1.2 Anti-BLV antibody detection

Anti-BLV antibodies were detected in serum samples by enzyme-linked immunosorbent assay (ELISA) performed using an anti-BLV antibody ELISA kit (JNC, Tokyo, Japan) according to the manufacturer's instructions.

#### 2.1.3 BLV DNA detection

After hemolysis of red blood cells with 0.83% ammonium chloride, white blood cells (WBCs) were isolated and DNA was extracted using QIAamp DNA Mini Kit (51304, Qiagen, Hilden, Germany) following the manufacturer's instructions. WBCs DNA concentration was measured by using a spectrophotometer, NanoDropLite (Thermo Fisher Scientific, Waltham, MA, USA). Nested polymerase chain reaction (nested PCR) was carried out to amplify the envelope or pX region of BLV [1,2]. Nested PCR was performed in a total reaction volume of 20 µL containing 0.5 U of DNA polymerase from GoTaq Hot Start Green Master Mix (M5122, Promega, Madison, WI, USA) or SapphireAmp Fast PCR Master Mix (RR350A, Takara Bio, Kusatsu, Japan), 0.5 µmol/L of forward and reverse primers, and 1 µL of extracted DNA (100–400 ng). The thermal cycling conditions were 95 °C for 2 min, 35 cycles of 94 °C for 45 s, 62 °C for 30 s, 72 °C for 30 s, and lastly 72 °C for 4 min.

#### 2.1.4 BLV copy number measurement

BLV copy number was measured from extracted WBCs DNA by quantitative real-time PCR. The reaction mixture contained 10 µL of THUNDERBIRD Probe qPCR Mix (A4250K, Toyobo, Osaka, Japan), 0.3 µL of CoCoMo-BLV Primer/Probe (A803, Riken Genesis, Tokyo, Japan), 5 µL of DNA template, and PCR grade water was added up to 20 µL. For the proviral quantification, BLV BoLA-DRA gene Plasmid DNA was used from the kit (A804, Riken Genesis) and BLV proviral DNA was measured by a Thermal Cycler Dice Real Time System III (TP970, Takara Bio) according to the manufacturer's instructions. BLV copies of >30,000/10^5^ WBCs DNA was considered as HPL in BLV-infected cattle (Table 1).

#### 2.1.5 LDH isozymes measurement

LDH isozymes were measured by Hydrasys 2 Scan (Sebia, Lisses, France) using Hydragel 7 ISO-LDH (PN4112, Sebia), conducted by an assigned clinical laboratory testing company (Fujifilm Vet Systems, Tokyo, Japan). BLV-infected cattle with LDH 2 + 3 >30% was considered as high LDH (HLDH) (Table 1).

### 2.2 Milk sampling

#### 2.2.1 Sample collection

Milk samples were collected from three BLV-infected cattle with HPL+HLDH and three uninfected cattle based on the hematological parameters (Table 1). The milk samples were placed in a cool box to maintain the temperature and were transported quickly to the laboratory.

#### 2.2.2 Isolation and detection of milk EVs

Milk EVs were isolated as previously described [3,4]. Milk EV morphological features were detected by TEM analysis [5].

#### 2.2.3 Proteomic analysis of milk EVs

For the proteomic analysis of milk EVs, a comprehensive nanoLC-MS/MS method was performed by using an UltiMate 3000 RSLC nano System (Thermo Fisher Scientific) coupled with Q Exactive mass spectrometer (Thermo Fisher Scientific) as described previously [6]. All procedures of proteomic analyses were carried out by an assigned company (Hakarel, Osaka, Japan). The identified proteins were further analyzed by using a proteomic software Scaffold-DIA (Portland, OR, USA) considering the false discovery rate >1%. The obtained MS/MS spectra was evaluated based on the UniPort database for *Bos taurus* (https://www.uniprot.org/proteomes/UP000009136) for annotation of the accession number of each identified EV protein. The proteins without a gene name or those having a dual gene name were omitted from our datasets.

## Ethics Statement

The experiments were conducted in accordance with all relevant guidelines and regulations of the Gifu University Animal Care and Use Committee (Approval numbers: 17046 and 2019–234).

## Funding

This study was supported by a regulatory research project for food safety, animal health, and plant protection (JPJ008617.17935709) granted by the Ministry of Agriculture, Forestry, and Fisheries, Japan, and by the 10.13039/100010742OGAWA Science and Technology Foundation, the Morinaga Foundation for Health and Nutrition, and the Sasakawa Scientific Research Grant from The 10.13039/501100007807Japan Science Society.

## Declaration of Competing Interest

The authors do not have any competing financial interests or personal relationships to declare that could influence the work reported herein.
